# Lethal outcome of granulomatous acanthamoebic encephalitis in a man who was human immunodeficiency virus-positive: a case report

**DOI:** 10.1186/s13256-018-1734-8

**Published:** 2018-07-12

**Authors:** Stefanie Geith, Julia Walochnik, Franz Prantl, Stefan Sack, Florian Eyer

**Affiliations:** 1 Division of Clinical Toxicology & Poison Control Centre Munich, Department of Internal Medicine II, TUM School of Medicine, Technical University of Munich, Ismaninger Str. 22, 81675 Munich, Germany; 2 Medical University of Vienna, Center for Pathophysiology, Infectiology and Immunology, Institute of Specific Prophylaxis and Tropical Medicine, Kinderspitalgasse 15, 1090 Vienna, Austria; 3 Institute of Pathology, Academic Clinic Munich-Schwabing, Kölner Platz 1, 80804 Munich, Germany; 4 Department of Cardiology, Pneumology and Internal Intensive Medicine, Academic Clinic Munich-Schwabing, Kölner Platz 1, 80804 Munich, Germany

**Keywords:** *Acanthamoeba* species, GAE, HIV, Miltefosine treatment

## Abstract

**Background:**

*Acanthamoeba* species can cause disseminating infections in immunocompromised individuals.

**Case presentation:**

Here, we report a case of granulomatous acanthamoebic encephalitis with a lethal outcome in a 54-year-old German man who was human immunodeficiency virus-positive. The diagnosis was based on symptoms of progressive neurological deficits, including sensorimotor paralysis of his right leg and deteriorating alertness. Due to the rapid course and rather late diagnosis of the infection, effective treatment could not be applied and he died 12 days after hospital admission.

**Conclusions:**

To the best of our knowledge, this is the second case of granulomatous acanthamoebic encephalitis reported within Germany. Our case highlights the importance of early diagnosis of granulomatous acanthamoebic encephalitis to prevent fatal outcome.

## Background

*Acanthamoeba* species are free-living amoeboid single-cell organisms that naturally occur in water and soil, but can also be found in human-made habitats all over the world [1–5]. *Acanthamoeba* species are known as causal agents of disseminating infections in immunocompromised individuals (for example, human immunodeficiency virus (HIV) positive, immunosuppressive therapy), including granulomatous acanthamoebic encephalitis (GAE) [6–8]. GAE is a rare but mostly fatal disease [6, 9]. Furthermore, *Acanthamoeba* species can cause so-called *Acanthamoeba* keratitis; *Acanthamoeba* keratitis mainly occurs in contact lens wearers and increasing casualties have been reported in the past decades [10, 11].

## Case presentation

In December 2010, a 54-year-old German man presented to our hospital with suspected cerebral toxoplasmosis. HIV infection had been diagnosed in 1995. He had received a triple-combination highly active antiretroviral therapy (HAART) of lopinavir, lamivudine, and tenofovir, which was stopped in 2009 due to intolerable side effects (diarrhea, nausea).

Over a period of 5 days, progressive neurological deficits including sensorimotor paresis of his right leg and deterioration of alertness occurred.

On clinical and neurological examination, he showed high-grade flaccid paralysis of his right lower limb with preserved muscle proprioceptive reflexes and positive Babinski sign.

**Fig. 1:**
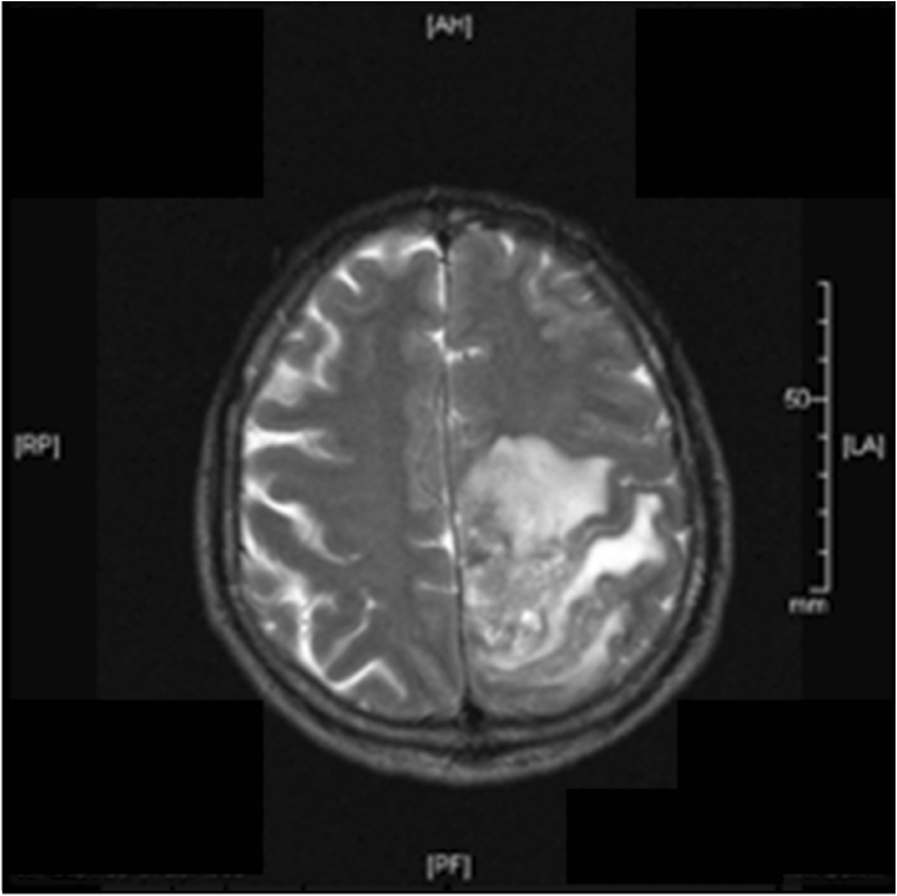
Magnetic resonance imaging on day 6 with pronounced perifocal edema in the left parieto-occipital region

Magnetic resonance imaging (MRI) on day 1 revealed a periventricular hyperintense lesion with perifocal edema in the left parieto-occipital region which continued to progress as shown in imaging on day 6 (Fig. 1).

**Table 1:** Results of diagnostics in cerebrospinal fluid, blood, serum, and bronchoalveolar lavage

Disease/Pathogen	Test	Result
Human immunodeficiency virus	Liquor, PCR Serum, PCR	Positive (670,000 copies/mL) Positive (2,200,000 copies/mL)
Toxoplasmosis	Immunohistochemistry	Negative
Measles	Liquor, Serum, IgG Serum, IgM	Negative Positive (IgG 1900 U/L) Negative
*Borrelia*	Liquor, IgG	Negative
Syphilis	Liquor, TPPA	Negative
FSME	Liquor, IgG Serum, IgG/IgM	Negative Both negative
*Cryptococcus*	Liquor, antigen screen	Negative
HSV 1/2	Liquor, DNA BAL, DNA	Negative Positive
VZV (herpes zoster)	Liquor, PCR	Negative
CMV	Liquor, PCR BAL, PCR	Negative Negative
EBV	Liquor, PCR	Negative
JCV (human polyomavirus)	Liquor, PCR BAL, PCR	Negative Negative
HCV	Serum, PCR	Negative
*Enterococcus*	BAL	Positive

*BAL* bronchoalveolar lavage, *CMV* cytomegalovirus, *EBV* Epstein–Barr virus, *FSME* tick-borne encephalitis, *HCV* hepatitis C virus, *HSV* herpes simplex virus, *JCV* John Cunningham virus, *PCR* polymerase chain reaction, *TPPA Treponema pallidum* particle agglutination assay, *VZV* varicella zoster virus

Blood laboratory values on day 1 revealed leukopenia (3.4/nL) and thrombocytopenia (101/nL).

No fungi, viruses (except HIV-1), or bacteria were detected in blood and cerebrospinal fluid cultures nor in serologic tests and polymerase chain reaction (PCR; Table 1). Prophylactic antibiotic treatment (antifungal, antiviral, antibacterial, and antiprotozoal) was administered as listed in Table 2. Immunocytology of cerebrospinal fluid on day 5 showed a reduced absolute lymphocyte count (640/μL), reduced T-helper cells (CD3, 365/μL), and a pathologic CD4/CD8 ratio.

**Table 2:** Initial antibiotic treatment

Medication	Daily dose	Route of administration
Pyrimethamine	37.5 mg	Orally
Fluconazole	2 × 100 mg	Intravenously
Clindamycin	1800 mg	Intravenously
Ceftriaxone	2 g	Intravenously
Aciclovir	3 × 750 mg	Intravenously
Meropenem	3 × 1 g	Intravenously

**Fig. 2:**
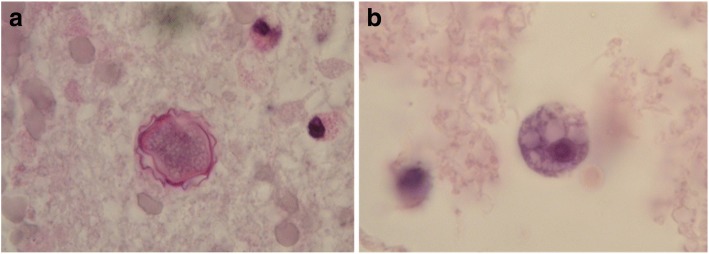
*Acanthamoeba* species. **a** Cyst with periodic acid–Schiff staining and **b** rounded trophozoite with hematoxylin and eosin staining (× 1000)

**Fig. 3:**
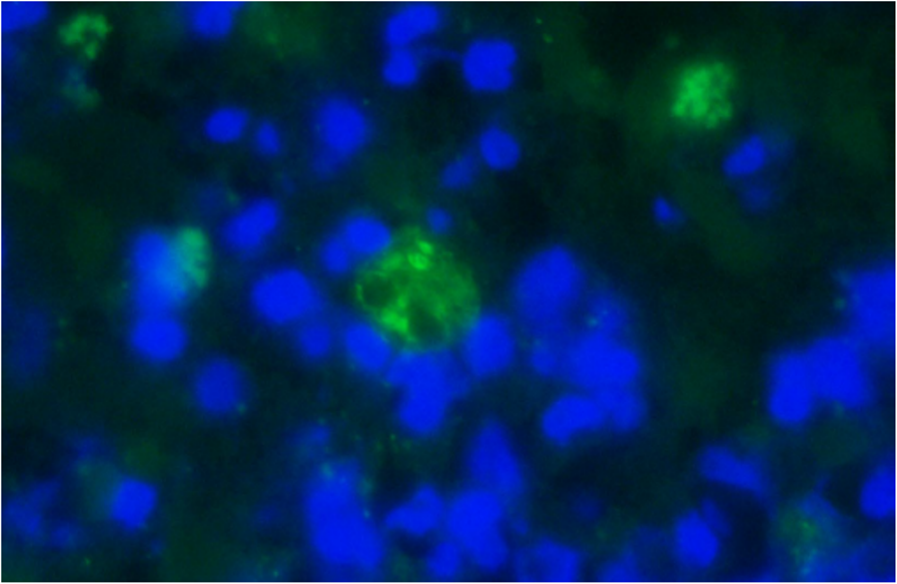
Immunostained *Acanthamoeba* trophozoite with characteristic nucleus and prominent contractile vacuole (Multichannel × 400)

Due to pathologic MRI findings, a stereotactic biopsy was performed on day 9. Histopathological results obtained on day 12 showed extensive tissue necrosis with mixed inflammatory infiltrates. Cysts of *Acanthamoeba* species were detected in periodic acid–Schiff (PAS) and Grocott stainings of brain specimens. Mononuclear trophozoites could be identified in hematoxylin and eosin (HE) and PAS stainings (Fig. 2). Furthermore, additional immunohistochemical staining was performed using an antibody specific to *Acanthamoeba* species (from rabbits immunized with *Acanthamoeba* genotype 4; Fig. 3).

Electroencephalography (EEG) on day 6 showed a lesion located in his left frontotemporal region with epileptic patterns in the left parietal lobe. He developed relapsing tonic-clonic seizures which normalized by day 8 following anticonvulsive therapy with valproic acid, methohexital, levetiracetam, and clonazepam.

MRI on day 11 revealed a new and massive ubiquitous subarachnoid hemorrhage, a beginning compression, a generalized cerebral swelling, and, an expanding left parietal periventricular lesion.

Due to the unfavorable prognosis, we, in agreement with his relatives, took no further intensive care measures. He died on day 12 after hospital admission.

Since histopathology did not reveal GAE before day 12, a specific treatment against GAE (for example, miltefosine-based combination therapy) had not been initiated.

## Discussion and conclusions

Infections with *Acanthamoeba* species are rare; hence, clinicians, pathologists, and clinical microbiologists are generally unfamiliar with these diseases. The vast majority of cases of GAE reported in the literature have been diagnosed postmortem [12, 13]. To the best of our knowledge, this is the second case of GAE reported within Germany.

The current case emphasizes the importance of early diagnosis of GAE. Microscopy of centrifuged fresh cerebrospinal fluid is recommended to diagnose *Acanthamoeba* trophozoites, yet these may be misdiagnosed as macrophages. Histological staining enables a clear differentiation of trophozoites from other cells [13]. Today, PCR is the method of choice for rapid, specific, and sensitive detection of *Acanthamoeba* species in clinical samples and also allows genotype identification [14] as well as diagnosis from formaldehyde-fixed samples [15, 16]. Early diagnosis and specific treatment is only possible if an infection with *Acanthamoeba* species is suspected early [13].

Today, there is no standard regimen for the treatment of GAE, but several successfully treated cases have been reported. For example: a patient with acquired immunodeficiency syndrome (AIDS) was treated with a combination of fluconazole and sulfadiazine [17]; two immunocompetent children received trimethoprim-sulfamethoxazole, rifampicin, and ketoconazole [18]; another immunocompetent woman was treated with fluconazole, rifampicin, and metronidazole [13]; a young immunocompromised man with underlying tuberculosis was treated with miltefosine, amikacin, and tuberculostatic drugs [8]; and, another young immunocompetent man was treated with rifampicin, moxifloxacin, and fluconazole [19]. The Centers for Disease Control and Prevention (CDC) recommends administration of miltefosine based on 26 reported cases in which a miltefosine-including regimen seemed to offer a survival advantage [20].

Our patient was prophylactically treated with antibiotics, covering antifungal, antiviral, antibacterial, and antiprotozoal activity. He did not receive a miltefosine-based combination therapy. As pointed out earlier, most reported cases of GAE have been diagnosed postmortem [12] and all successfully treated cases were detected early and mainly by chance. Therefore, both awareness and an early and specific diagnosis followed by an immediate start of a miltefosine-based treatment seem of crucial importance for the successful treatment of GAE. *Acanthamoeba* species should be considered in patients with unclear encephalitis, particularly in immunocompromised patients.

## Abbreviations

AIDS: Acquired immunodeficiency syndrome
CDC: Centers for Disease Control and Prevention
EEG: Electroencephalography
GAE: Granulomatous amoebic encephalitis
HAART: Highly active antiretroviral therapy
HE: Hematoxylin and eosin
MRI: Magnetic resonance imaging
PAS: Periodic acid–Schiff
PCR: Polymerase chain reaction
